# The impact of self-compassionate mindfulness on online learning behavioral engagement of international students during COVID-19: Positive emotion and self-improvement motivation as mediators

**DOI:** 10.3389/fpsyg.2022.969657

**Published:** 2022-09-15

**Authors:** Junmei Chen, Guoyao Lin, Yong Lyu

**Affiliations:** ^1^Faculty of Psychology, Tianjin Normal University, Tianjin, China; ^2^College of Chinese Language and Culture, Huaqiao University, Xiamen, China; ^3^Fujian Key Laboratory of Applied Cognition and Personality, School of Educational Science, Minnan Normal University, Zhangzhou, China

**Keywords:** self-compassionate mindfulness, online learning behavioral engagement, positive emotion, self-improvement motivation, COVID-19

## Abstract

Focusing on the domain of self-compassion, this study explored the promotion mechanism of online learning behavioral engagement (OLBE) of international students in China under COVID-19. Positive emotion and self-improvement motivation were selected as mediators. Participants were 606 international students from 8 countries who were studying online in their own countries due to the international travel restriction of COVID-19. Results showed positive emotion and self-improvement motivation completely mediated self-compassionate mindfulness (SCM) and OLBE of international students. Positive emotion and self-improvement partially mediated SCM and OLBE of international students respectively. Students with higher SCM engage with online learning more in that they possess more positive emotion and self-improvement motivation. This study suggested that SCM may facilitate OLBE via positive emotion and effective self-improvement motivation.

## Introduction

China has the largest number of international students in Asia. In 2018, 492,200 international students were studying in China (Zhao, 2020). Due to the international travel restriction of COVID-19, the majority of international students are stranded in their own countries after 2019. Giving Indonesian students for example, before the epidemic, there were 15,760 Indonesian students studying in China. From January to February 2020, 14,408 students had returned to Indonesia due to the epidemic, while only 1,352 students were still studying on Chinese university campuses (Min, 2021). Students stranded in their home countries are forced to study online. The stress caused by the epidemic and the sudden change of learning style from offline to online had a significant impact on students’ mental health (Cao et al., 2020). More than three-quarter students considered that online learning is not a positive experience (Min, 2021). Students reported feeling stressed, bored and anxious while studying online during the pandemic (Espino et al., 2021). Increasing Students’ engagement in online learning during the pandemic has become an academic concern.

Kuh (2009) defined learning engagement as time and efforts that students invested in learning activities. Fredricks et al. (2004) divided learning engagement into three dimensions: behavioral engagement, cognitive engagement, and emotional engagement. Some scholars believe that learning behavioral engagement is an important branch of learning engagement. Learning behavioral engagement refers to the time, energy, and effort students invest in learning activities, which can be observed as explicit behavioral performance (Li et al., 2016). For example, attend class on time, listen carefully, answer teachers’ questions actively, submit homework on time, and do not give up when encountering problems. Learning behavioral engagement can be summarized into three categories: actively implementing relevant regulations and requirements, without any violation or destructive behavior (such as skipping class); performance in study or academic tasks, such as effort, persistence, concentration, questioning, participation in discussions, etc.; participation in extracurricular and school-related activities, such as sports or school administration (Fredricks et al., 2004). Research shows that learning behavioral engagement can influence and predict subsequent emotional and cognitive engagement (Li and Lerner, 2013). Learning behavioral engagement is the premise of skill development, positive social interaction, and emotional engagement (Wu and Qi, 2018). Timely feedback and intervention on learning behavioral engagement can effectively improve learning performance. In the long run, learning behavioral engagement can predict learners’ academic failure, graduation, and academic adaptability (Wu and Qi, 2018). Therefore, this article selects “online learning behavioral engagement (OLBE)” as the dependent variable. OLBE refers to Students’ active implementation of relevant regulations and requirements in online learning, as well as their independent learning behaviors in learning and academic tasks, which can be observed as explicit behaviors. Hereinafter refers to as “OLBE.”

### Self-compassionate mindfulness and online learning behavioral engagement

Self-compassion is described as the tendency to be kind, loving, and understanding regarding oneself in the middle of one’s pain and shortcomings, as opposed to being self-critical and over-identifying with negative emotions (Neff, 2003b). Self-compassion is comprised of three (bipolar) components: self-kindness (vs. self-judgment); common humanity (vs. *i*solation); mindfulness (vs. over identification).

During the pandemic, academics have conducted some research on the topic of self-compassion and COVID-19. In Turkey, Deniz (2021) demonstrated that self-compassion not only enhanced citizens’ tolerance for uncertainty, buffering the effects of negative events on happiness but also directly reduced the fear of COVID-19. In Spain, researchers selected 917 participants to study the mental health of citizens during the lockdown and concluded that self-compassion could make individuals less susceptible to anxiety, depression, and stress (Gutiérrez-Hernández et al., 2021). Also in Spain, self-compassion and mindfulness have been found to reduce burnout and compassion exhaustion among healthcare workers during the pandemic (Ruiz-Fernández et al., 2021). At the epidemic peak in Hong Kong, China, the negative side of self-compassion was found to increase citizens’ psychological stress, while the positive side enhanced individuals’ abilities to perceive more benefits from the epidemic (Lau et al., 2020). Chinese researcher Liu and Wu (2020) has also proposed the mechanism and intervention strategies of self-compassion for negative post-traumatic psychological reactions caused by the epidemic.

The preceding research examined the effect of self-compassion on emotional health and the wellbeing of people in the context of the world pandemic. To date, there was no report of self-compassion for students who were forced to take online classes due to the pandemic. Metawee (2020) reported that self-compassion could promote online learning self-efficacy, and finally promote academic flow among students with hearing impairments. However, so far, very little is known as to whether self-compassion promotes online learning engagement. Previous research studies have concluded that self-compassion had a good correlation with intrinsic motivation and learning engagement of nursing students (Kotera et al., 2021), and mindfulness positively predicted job engagement (Malinowski and Lim, 2015; Li and Shen, 2019; Lv et al., 2021). By enhancing the experience of individual immersion and concentration, mindfulness can help the employee focus on the present moment, and improve their understanding of internal and external events, thus they can better understand the meaning of the work itself and participate in the work in a positive and focused state. In terms of learning engagement, Gao (2020) discovered a substantial association between the level of mindfulness of senior high school students and their learning engagement, among which mindfulness had the greatest predictive effect on emotional engagement.

The dimension of self-compassion also includes mindfulness, which is called self-compassionate mindfulness (SCM) by Wang et al. (2019), and it is a core element of self-compassion (Neff and Dahm, 2015). Mindfulness reflects an individual’s non-judgmental awareness of the present situation in general (Bishop et al., 2004; Kabat-Zinn, 2021). While SCM is narrower in scope because it focuses more on the awareness of pain in balance when an individual suffers difficulties or setbacks (Goff, 2011). The SCM scale includes items such as “When something upsets me, I try to keep my emotions in balance.” The COVID-19 pandemic is one of the greatest dilemmas encountered in human history. Being forced to take classes online in a small room can be a frustrating and even painful experience for students. What’s more, they have to deal with negative emotions and drastic changes in their environment caused by COVID-19. SCM is expected to help them stay calm and take a balanced view of such situations. Therefore, in this study, SCM was selected as an independent variable. We speculated that it had a positive predictive effect on the OLBE of international students in China during the epidemic.

### Mediating role of self-improvement motivation and online learning behavioral engagement

Self-improvement motivation refers to the psychological process of an individual’s efforts to make the self-perception more positive to gain a sense of progress and growth (Sun and Li, 2012). Self-compassion is a way of relating to the experiencer who is suffering (Neff and Dahm, 2015). It is characterized by increased self-kindness and less self-judgment, increased common humanity, and less isolation, as well as greater mindfulness and less overindulgence in negative thoughts and emotions (Neff, 2003a). Individuals with high levels of self-compassion can alleviate negative emotion caused by painful events (Leary et al., 2007). While buffering negative emotions, self-compassion is not content with the *status quo* but encourages individuals to constantly improve themselves in difficult situations. Breines and Chen (2012) believed that participants in a state of self-compassion exhibited more positive thoughts toward personal weakness, indicated increased motivation to pay for moral violations, spent additional time learning a challenging task, and demonstrated a tendency for upward social comparisons. In conclusion, self-compassion was connected with increased motivation for self-improvement in a variety of ways and among people. There were also many studies showing that individuals with a higher level of mindfulness exhibited more autonomic motivational behaviors (Levesque and Brown, 2007; Li and Shen, 2019). Kroon et al. (2015) regarded mindfulness as a personal resource, arguing that people with mindful thinking gain a sense of control when facing difficulties and challenges, improve their ability to adapt to the work environment, stimulate their intrinsic motivation, and thus improve their work performance. It can be concluded that SCM positively predicts self-improvement motivation.

In terms of motivation and learning engagement, learning motivation had a substantial positive influence on learning engagement (Zhang, 2016). Lv and Yang (2013) found that learners’ intrinsic interest, desire for cultural exchange, as well as personal development could stimulate their highly engaged learning behaviors. Studies on online learning engagement found that academic self-efficacy, intrinsic motivation, and connected classroom atmosphere had significant impacts on the online learning engagement of college students (Wei et al., 2019). Some researchers examined the effects of autonomous and controlled motivation on online learning engagement and found that autonomous motivation positively predicted their learning engagement behavior while controlling motivation negatively predicted online learners’ academic engagement (Gao et al., 2015).

In conclusion, this study hypothesizes that self-improvement motivation mediates SCM and OLBE.

### Mediating role of positive emotion and self-compassionate mindfulness

Self-compassion is positively associated with positive affect (Neff et al., 2007; Rainbow et al., 2022). Both the total score of self-compassion and SCM subscale are positively associated with subjective wellbeing (Hu, 2013). The Broaden-and-Build model of positive emotions (Isgett and Fredrickson, 2004) suggests that the increase in positive emotions would result in the broadening of one’s momentary-thought-action repertoire which, in turn, would lead to increased personal resources and subjective wellbeing. Therefore, we expect that SCM is positively associated with positive emotion. What is more, SCM is a narrower kind of mindfulness which focuses on balanced awareness of negative thoughts and feelings (Neff and Dahm, 2015). Mindfulness meditation training processes attention through a purposeful, open, and non-judgmental attitude. Individuals are educated to retain a transient attitude toward all psychological occurrences, which might result in a high tolerance for unpleasant interior states, thereby increasing happiness. Mindfulness expands attention and strengthens cognitive flexibility, redefines or constructs stressful events, and ultimately triggers positive emotion that relieves stress (Chen et al., 2011). Thus, we predict that SCM would predict positive emotion.

The occurrence and development of motivation involve many physiological and psychological variables, among which emotion plays a central role. Emotion not only restricts the motivation expression of other physiological and psychological variables, but also constitutes a unique motivation power. Meanwhile, the development of emotion directly affects the development of motivation (Qiao, 1993). Pentaraki and Burkholder (2017) showed that emotions (including pride, hope, relief, anger, anxiety, shame, despair, and boredom) were considerably related to academic success, learning strategies, academic motivation, self-regulation, and value evaluation. It is hypothesized that positive emotions mediate SCM and self-improvement motivation.

### The current study

**FIGURE 1:**
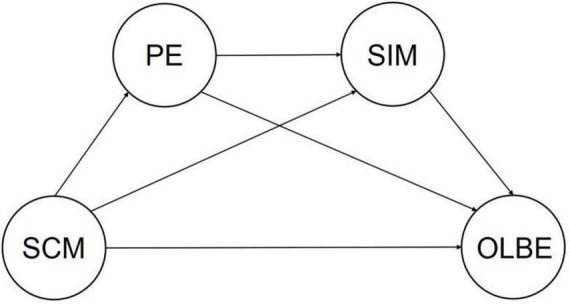
Research model.

Due to the impact of the new coronavirus, most international students in China have not been able to return to campuses to continue their studies. In the great dilemma shared by human beings, individuals need to empathize with others as well as with themselves. However, so far, no research has been conducted on self-compassion and online learning engagement, nor has there been any research on the online learning engagement of international students in China. Based on the above analysis, this study proposes the following hypotheses (Figure 1):

**H1:** Self-improvement motivation mediates SCM and OLBE;

**H2:** Positive emotion mediates SCM and OLBE;

**H3:** Positive emotion and self-improvement motivation play a chain mediating role between SCM and OLBE.

## Materials and methods

### Participants

The method of simple random cluster sampling was adopted, 637 international students from 3 universities in Fujian Province were selected as subjects, and 606 valid questionnaires were effectively recovered. The ages of the participants ranged from 17 to 34 years, with an average age of 20.54years (SD = 1.62). Among them, 144 were male students, 462 were female students, 176 were 1st-year students, 215 were 2nd-year students, 205 were 3rd-year students, and 10 were 4th-year students. Details about the country distribution of the students are shown in Table 1.

**TABLE 1:** Characteristics of participants.

Characteristics		n	Percentage (%)
Gender	Male	144	23.7
	Female	462	76.1
Year	First year	176	29.0
	Second year	215	35.4
	Third year	205	33.8
	Fourth year	10	1.6
Country	Thailand	237	39.11
	Indonesia	246	40.59
	Philippine	47	7.76
	Laos	44	7.26
	Myanmar	22	3.63
	Peru	3	0.5
	Pakistan	2	0.33
	Vietnam	5	0.83
Age	17–21	499	82.35
	22–26	94	15.51
	27–34	13	2.14
Total		606	100

### Measures

A bilingual survey was conducted because participants may have various levels of English proficiency. The survey included English items from the original scales, as well as a Chinese version translated and back-translated by the researcher and a lecturer in translation.

#### Self-compassion

Self-compassion was assessed using the self-compassion scale (SCS; Neff, 2003a, adapted into China by Gong et al., 2014.) The SCS consists of three subscales: self-kindness, common humanity, and mindfulness. The items were scored on a 5-point scale, with higher scores indicating a higher level of self-compassion. Sample items include “When things go wrong, I can understand that frustration is part of the life experience,” and “When painful things happen, I try to look at them objectively.” The entire scale obtained a Cronbach coefficient of 0.77, whereas the subscale of mindfulness had a Cronbach coefficient of 0.737. In this study, only the mindfulness subscale was selected, and its Cronbach coefficient was 0.767 in this study.

#### Positive emotion

Positive emotion was measured using the positive affect subscale in the positive and negative affect scale (PANAS, Watson et al., 1988). Respondents used a 5-point Likert scale (from 1 = totally disagree to 5 = strongly agree). The time indication given was “here and now.” Sample items include “Inspired,” “Proud” etc. The higher the score, the higher the level of positive emotion. This scale has been demonstrated to be reliable. The Cronbach coefficient of the positive affect scale was between 0.84 and 0.87. The Cronbach coefficient of PANAS Chinese version was 0.83, and the Cronbach coefficient of the positive emotion scale was 0.85 (Huang et al., 2003). In this study, the Cronbach coefficient of the positive affect scale was 0.76.

#### Online learning engagement

Fredricks et al. (2005, 2004) developed online learning engagement (Sun and Rueda, 2012). It is a scale consisting of three types of engagement: behavioral engagement, emotional engagement, and cognitive engagement. The behavioral subscale of online learning engagement includes five items such as “I complete my homework on time,” “I check my homework for mistakes.” The items were scored on a 5-point scale, with higher scores indicating a higher level of. Confirmatory factor analysis was performed. The validity index is shown in Table 2. In this study, the Cronbach coefficient of the scale was 0.788.

**TABLE 2:** Confirmatory factor analysis results of OLBE.

Model fitting index of OLBE scale								

χ^2^/df	CFI	RMSEA	NFI	GFI	PGFI	AGFI	IFI	TLI
2.835	0.995	0.048	0.992	0.995	0.199	0.977	0.995	0.984

#### Self-improvement motivation

The self-improvement motivation is a single dimension self-rating scale of Breines and Chen (2012). The 7-item was rated on a 7-point scale with a Cronbach coefficient of 0.80. The higher the score, the higher the level of self-improvement motivation. Sample items include “I feel capable of making positive changes,” or “ I would like to discover new strategies for improving myself.” In this study, the Cronbach coefficient of the scale was 0.887.

## Results

### Strategy for data analysis

Firstly, descriptive statistics, Pearson correlation analysis, and common method bias analysis were performed with SPSS 22.0. Secondly, we used M-PLUS 8.0 to access the model based on the fit indices of the root mean square error of approximation (RMSEA), goodness-of-fit index (GFI), the Tucker-Lewis Index (TLI), comparative fit index (CFI), and chi-square. The model is deemed acceptable when the fit indices meet the following criteria: CFI > 0.90, RMSEA < 0.08, and χ*^2^/df* < 3. Finally, we conducted a bootstrap method with 1,000 resamples to test the direct and indirect effect of SCM and OLBE. The premise of mediation analysis is that there is a clear relationship between variables, that is, the correlation coefficient or regression coefficient should be significant, the correlational results between all the variables are all significant, therefore it meets the condition of mediation analysis. Because the two mediating variables emotion and motivation are closely related, we chose the Serial Multiple Mediator Model. The effect is considered significant if the CIs do not contain zero.

### Common method bias

All data were self-reported by the participants, raising the possibility of a common method bias in the measurement. The following is how we controlled for such bias. First, participants responded to the scales anonymously and were assured that their responses would be kept confidential and used solely for scientific study. Second, for statistical control, we used Harman’s one-factor test (Zhou and Long, 2004) which examined all variables’ items using unrotated principal component factor analysis. The results demonstrate that the variation explained by the first factor is 23.41%, which is less than the crucial value of 40%, suggesting the absence of a significant common method bias in this investigation.

### Correlation analysis

Table 3 displays the findings of the descriptive statistics and correlation analyses. OLBE had a significantly positive correlation with SCM, positive emotion, and self-improvement motivation. Positive emotion had a significant correlation with SCM and self-improvement motivation, thereby meeting the condition of mediation analysis.

**TABLE 3:** Means, standard deviations, and bivariate correlations.

Variable	1	2	3	4	5
1. Age					
2. Self-compassionate mindfulness	–0.45				
3. Positive emotion	–0.044	0.485**			
4. Online learning behavioral engagement	–0.062	0.442**	0.498**		
5. Self-improvement motivation	–0.023	0.452**	0.450**	0.490**	
M	20.54	3.37	3.40	3.70	5.38
SD	1.62	0.69	0.73	0.75	0.98

N = 606. **p < 0.01.

### Mediation effect test

**FIGURE 2:**
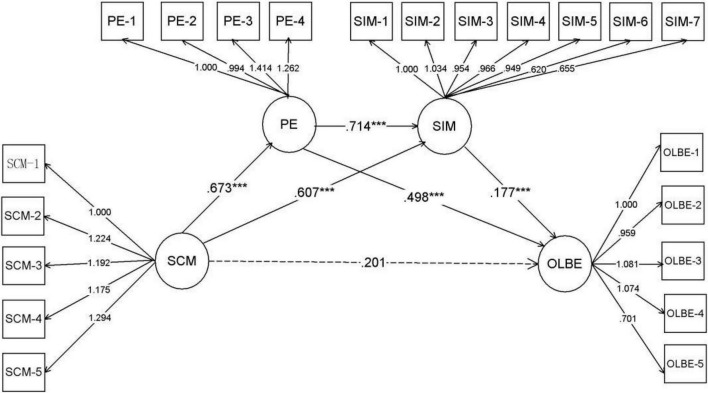
The mediation model (*N* = 606). SCM-1–SCM-5 are five aspects of self-compassionate mindfulness; PE-1-PE-4 are four aspects of positive emotion. SIM-1–SIM-7 are seven aspects of self-improvement motivation; OLBE-1–OLBE-5 are five aspects of online learning behavioral engagement. Standardized path coefficients and standardized factor loading are presented. ***p < 0.001.

We used the M-PLUS 8.0 to confirm the mediating model, and the fitting indexes of the whole model were obtained as follows: χ*^2^* = 483.567, χ*^2^/df* = 2.642, RMSEA = 0.052,

CFI = 0.942, TLI = 0.933, indicating that the data fits well with the model (Figure 2).

The results indicated that while the total effect of SCM on OLBE was significant [βtotal = 0.528, SE = 0.092, 95%CI (0.369, 0.727)], the direct effect was not [βtotal = 0.201, SE = 0.11, 95%CI (−0.014, 0.417)]. Overall, the two mediators fully mediated the relationship between SCM and OLBE. The path coefficients in Figure 2 showed that SCM positively predicted positive emotions (β = 0.673, *P* < 0.001) and self-improvement motivation (β = 0.607, *P* < 0.001), while positive emotions positively predicted self-improvement motivation (β = 0.714, *P* < 0.001) and online learning behavior engagement (β = 0.498, *P* < 0.001). Self-improvement motivation positively predicted online learning behavior engagement (β = 0.177, *P* < 0.001). The Bootstrap method was used to verify the mediation effect of the model in Figure 2, and the results are shown in Table 4.

**TABLE 4:** Bootstrap results of each path coefficient of the model.

	Path	Effect	SE	95% CI	*P*
Mediating effect	scm→pe→sim→olbe	0.085**	0.029	[0.043, 0.16]	0.003
	scm→ sim →olbe	0.108**	0.04	[0.049, 0.212]	0.007
	scm→pe→olbe	0.335***	0.078	[0.199, 0.496]	0.000
Total mediating effect		0.528***	0.092	[0.369, 0.727]	0.000
Direct effect	scm→olbe	0.201	0.11	[−0.014, 417]	0.067

N = 606. **p < 0.01, ***p < 0.001.

## Discussion

To our knowledge, this is the first study investigating SCM in relation to OLBE during the novel coronavirus pandemic period. To a certain extent, the study confirmed the hypothesis that positive emotion and self-improvement motivation exert a mediating influence on the relationship between SCM and OLBE. The more mindful a student was, the higher OLBE tended to be. This study found that SCM had no direct impact on OLBE, which was similar to Malinowski and Lim’s (2015) discovery that positive emotions, such as hope, and optimism completely mediated between mindfulness and work engagement. Though mindfulness in Malinowski and Lim’s (2015) article was measured with the Five Facets of Mindfulness Questionnaire (FFMQ), research showed that self-compassion and mindfulness (measured with FFMQ) were significantly interrelated (*r* = 0.69; Hollis-Walker and Colosimo, 2011). López et al. (2016) considered that mindfulness and self-compassion are strongly linked constructs that go hand in hand. SCM is a narrower kind of mindfulness focusing especially on suffering and setbacks. While individuals suffer, self-compassion can act as a mood stabling strategy, decrease negative emotion, increase positive emotion, and finally adopt more adaptive behavior (Berking and Whitley, 2014). As universities shifted from offline to online due to COVID-19, learning engagement has been considered as a challenge (Zhang et al., 2021). This study may provide directions for improving the quality of online learning.

The mediating role of positive emotion between mindfulness and engagement was found in this study, which was consistent with the report by Pekrun et al. (2002). This study proposed a cognitive-motivational model of academic emotions, and pointed out that positive classroom academic emotions would promote Students’ learning engagement, while negative emotions (such as depression, sadness, anger, etc.) would reduce Students’ learning engagement. But the predecessors did not involve the field of online engagement. Transiting abruptly from offline to online instruction, more than three-quarter students had negative emotion. Research shows that anxiety, focus, and workload were strongly related, indicating the problems faced by students to maintain focus and do assignments on learning activities while being at home (Espino et al., 2021). Cultivating SCM may be a way to increase positive emotion in the online study during the pandemic. Individuals with high levels of mindfulness are happier, more optimistic, more curious, and more passionate (Neff and Dahm, 2015), and the feeling of happiness persists for the next 1, 3, and 6 months. When facing a dilemma, highly mindful people are able to take a step back, perceive things more peacefully and clearly, reflect the current situation “as it is,” generate more positive emotion. And according to the extension-construction theory, when positive emotions are improved, psychological capital such as hope and optimism will increase, thus enhancing the sense of involvement (Isgett and Fredrickson, 2004).

Most previous research focused on the mediating role of motivation on mindfulness and job engagement. The effect of motivation on mindfulness and behavioral engagement in online learning is still rare. This study found that similar to offline learning engagement (Elphinstone et al., 2021), mindfulness also enhanced online learning engagement by enhancing motivation. Under the background of COVID-19, students forced to study online have less time and depth of interaction with classmates and teachers, less peer comparison pressure and supervision of teachers, and less extrinsic motivation. Autonomous motivation has become an important factor in online learning engagement and academic performance. However, research shows that there was a considerable reduction in the academic motivation of students during the Covid-19 pandemic (Zaccoletti et al., 2020). Motivation plays an important role in the self-regulated environment of university study. Research indicated that higher levels of motivation are connected with greater learning engagement (Elphinstone et al., 2021). The mediating effect of self-improvement motivation between SCM and OLBE provides an alternative way to improve the online learning effect of international students in China.

This chain mediation shows that SCM increases SIM by elevating PE, which ultimately increased OLBE. This result extends the engagement research from the work environment to online learning. Zheng et al. (2018) reported that psychological flourishing strongly mediated mindfulness on work engagement. Promoting personal resources and positive emotion could help people to enhance psychological flourishing, and organizations to increase work engagement. SCM perceives oneself in a peaceful, open, accepting manner when one is in adversity and promotes positive emotional regulation. It also helps individuals leave enough room for the restless mind to return to peace, allow the individual to regain vitality and motivation, and engage in online learning with more positive actions. Since February 2020, the complexity of the epidemic in different countries has prevented a vast number of international students from returning to campus. The lengthy online time tests Students’ academic persistence. Anxiety, irritability, loneliness, worry, and other negative emotions, drag students away from the original intention of hard working. It is particularly urgent to cultivate positive emotion and maintain self-improvement motivation thus promoting OLBE.

## Limitations

The exclusive usage questionnaire survey and self-reported data collection technique are considered as one of the major limitations. To properly study the process from SCM- to OLBE, qualitative methodologies such as diary analysis may be used in future research. In addition, this study is a cross-sectional study, so no clear causal relationship between SCM and OLBE can be obtained. The causal relationship between SCM and OLBE needs to be clarified by longitudinal follow-up in the future. Finally, positive emotion and self-improvement motivation were regarded as mediators between SCM and OLBE, another point that deserve exploring is to investigate the relationship between SCM and other dimension of online learning engagement such as emotional and cognitive engagement.

## Conclusion

Emotion and motivation are important factors in online learning engagement. Compare with previous research, few studies have investigated the link between self-compassion and learning engagement. The findings of the current study extend our understanding of the relationship between SCM and OLBE by revealing the mediation role of PE and SIM. International students studying in China who have higher SCM also tend to be more positive and show a higher level of SIM, thus more engage in the online study during COVID-19. The result that PE and SIM completely mediated SCM and OLBE has offered a direction to improve the quality of online learning. It is of crucial importance for online learners to practice SCM during boring and lonely online study time.

## Data availability statement

The raw data supporting the conclusions of this article will be made available by the authors, without undue reservation.

## Ethics statement

The studies involving human participants were reviewed and approved by the Ethics Committee of Tianjin Normal University. The patients/participants provided their written informed consent to participate in this study.

## Author contributions

JC performed the questionnaire survey and wrote the manuscript. GL performed the data analyses. YL performed the analysis with constructive discussions. All authors contributed to the article and approved the submitted version.
